# Supplementary material for: Intermittent low-dose far-UVC irradiation inhibits growth of common mold below threshold limit value
Source: PLoS One. 2024 Jul 2;19(7):e0299421. doi: 10.1371/journal.pone.0299421 (PMC11218994; doi:10.1371/journal.pone.0299421)
Supplement: S1 Fig — DG18 agar plates were irradiated with either 0, 100 or 400 mJ/cm2 far-UVC light. Immediately after irradiation, P. candidum spores (3.4 × 10^6^ spores/mL) was seeded on the plates in 8 μL droplets with five droplets on each plate. The inoculated plates were incubated at 24°C for three days, and the diameter of the seeded droplets was measured before and after incubation. The procedure was repeated twice. The growth on the plates was found to be not significantly different (one-way ANOVA, p > 0.05), indicating that the DG18 agar plates were not affected by far-UVC irradiation. (DOCX) [file pone.0299421.s001.docx]

**S1 Fig. *Penicillium candidum* growth on pre-irradiated agar plates.** DG18 agar plates were irradiated with either 0, 100 or 400 mJ/cm^2^ far-UVC light. Immediately after irradiation, *P. candidum* spores (3.4 × 10^6^ spores/mL) was seeded on the plates in 8 µL droplets with five droplets on each plate. The inoculated plates were incubated at 24°C for three days, and the diameter of the seeded droplets was measured before and after incubation. The procedure was repeated twice. The growth on the plates was found to be not significantly different (one-way ANOVA, p > 0.05), indicating that the DG18 agar plates were not affected by far-UVC irradiation.
