# Supplementary material for: Intermittent low-dose far-UVC irradiation inhibits growth of common mold below threshold limit value
Source: PLoS One. 2024 Jul 2;19(7):e0299421. doi: 10.1371/journal.pone.0299421 (PMC11218994; doi:10.1371/journal.pone.0299421)
Supplement: S2 Fig — Ozone measurements were conducted in the boxes using the same setup described in the methods section “Inhibition of P. candidum growth from airborne spores by far-UVC light”, with the highest UV dose of 100 mJ/cm2 per 24 hours. Ventilation was on, and the humidity was set to 80%. A calibrated EZ-1Z (Scanion, Denmark), which measures ozone between 20–140 ppb, was placed at the bottom of the box in the same location as the agar plates. After reaching equilibrium, the ozone level was below the detection limit. As a positive control, the lamp was configured to be constantly on, resulting in the minimum detectable level of 20 ppb. The blue graph represents the ozone level when the lamp is always on, while the red graph represents the theoretical oscillating ozone levels during the duty cycle configuration. (DOCX) [file pone.0299421.s002.docx]

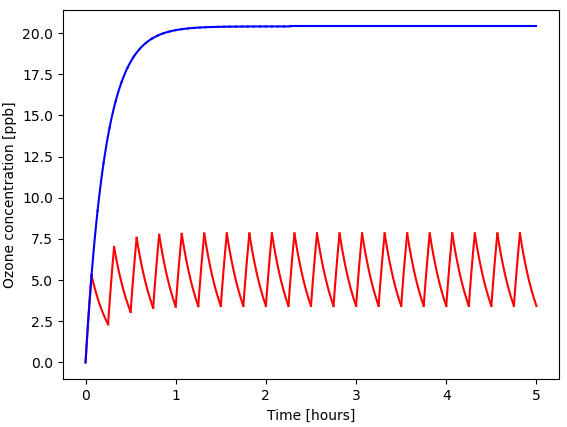


**S2 Fig. Measurements of ozone generated from far-UVC lamp.** Ozone measurements were conducted in the boxes using the same setup described in the methods section “Inhibition of *P. candidum* growth from airborne spores by far-UVC light”, with the highest UV dose of 100 mJ/cm^2^ per 24 hours. Ventilation was on, and the humidity was set to 80%. A calibrated EZ-1Z (Scanion, Denmark), which measures ozone between 20-140 ppb, was placed at the bottom of the box in the same location as the agar plates. After reaching equilibrium, the ozone level was below the detection limit. As a positive control, the lamp was configured to be constantly on, resulting in the minimum detectable level of 20 ppb. The blue graph represents the ozone level when the lamp is always on, while the red graph represents the theoretical oscillating ozone levels during on-off the duty cycle configuration.
